# Mechanism of curcumin in the prevention and treatment of oral submucosal fibrosis and progress in clinical application research

**DOI:** 10.1038/s41405-024-00268-7

**Published:** 2024-10-25

**Authors:** Rong She, Pu Xu

**Affiliations:** Haikou Affiliated Hospital of Central South University Xiangya School of Medicine, Haikou, 570208 China

**Keywords:** Mucositis, Dental treatments

## Abstract

**Introduction:**

Oral submucosal fibrosis is a potentially life-threatening oral disease that significantly impacts physiological functions such as speech and swallowing while also diminishing the quality of life for patients. Currently, the mainstream treatment for oral submucosal fibrosis in clinical practice involves invasive glucocorticoid drugs such as injection therapy. However, this method often leads to intraoperative pain, anxiety, fear, and poor medical experience due to associated side effects.

**Methods:**

There is an urgent need to actively explore new drugs and relatively noninvasive approaches for the treatment of oral submucosal fibrosis in order to enhance patients’ medical experience and compliance. This has become a focal point of attention in clinical research. After conducting an extensive literature search, it was discovered that curcumin, a natural polyphenolic compound, exhibits potent anti-tumor, anti-inflammatory, antioxidant, anti-metastatic and anti-angiogenic properties. Moreover, curcumin holds significant clinical potential in the prevention and treatment of various diseases such as oral submucosal fibrosis.

**Conclusion:**

This review presents a comprehensive elaboration encompassing the action mechanisms, biological activity, potential applications, and clinical characteristics of curcumin in the management of oral submucosal fibrosis, aiming to provide diagnostic insights and novel therapeutic perspectives for its prevention and treatment.

## Introduction

Oral submucosal fibrosis (OSF) is a chronic and insidious disease characterized by fibrotic changes in the connective tissue of the oral submucosa, with a potential risk of cancer. Its clinical symptoms mainly include mucosal burning sensation, pale mucosa, coarse fibrous feeling, and limited mouth opening. It is one of the most common precancerous lesions of oral squamous cell carcinoma (OSCC) [1]. The prevalence of OSF in China is primarily concentrated in the regions of Hunan, Hainan, and Taiwan. According to the World Health Organization, there exists a global burden of over 5 million cases of OSF [2]. The literature reports that the prevalence of OSF in China ranges from 0.96 to 3% [3].

According to the progression stage of OSF, there exist multiple therapeutic approaches, with the primary treatment methods falling into two distinct categories: conservative management and surgical intervention, conservative treatment includes physiotherapy, medical treatment. Although OSF is the preferred surgical treatment modality in severe cases, numerous studies have reported its postoperative outcome of deterioration, characterized by an escalation in fibrotic changes [4]. The main approach in conservative therapy for OSF involves hyperbaric oxygen treatment, which is associated with high costs, however, further clinical evidence is required to establish its efficacy in improving submucosal fiber cord and mouth opening in both cheeks and the soft palate [5].

Considering the noninvasive nature and high patient acceptance, drug therapy emerges as the predominant treatment modality for OSF. Currently, the predominant approach for clinical drug therapy involves local submucosal administration of glucocorticoids, such as triamcinolone, dexamethasone, methylprednisolone, and betamethasone. The administration of glucocorticoids effectively suppresses inflammation and inhibits the process of tissue fibrosis in patients. However, it fails to demonstrate significant efficacy in reversing fibrotic damage to tissues and restoring elasticity of oral mucosa. Moreover, patients experience intraoperative pain and exhibit poor postoperative compliance. Prolonged use of hormone drugs can lead to patient dependence, posing risks and challenges for clinical treatment [6–8]. Therefore, in order to better treat such diseases as OSF, actively looking for new drugs and new ways of administration for treating OSF has become a necessary theme. In recent decades, many clinical and randomized trials have not only evaluated the effectiveness of curcumin (CUR) on various autoimmune diseases [9]. Furthermore, CUR has demonstrated significant therapeutic efficacy in various fibrotic disorders [10]. In this review, we will comprehensively investigate the mechanism, biological activity, potential applications, and clinical characteristics of CUR in OSF, aiming to provide novel insights for clinical treatment and drug development.

## Overview of CUR

CUR, a natural polyphenolic compound found in the root of turmeric, is considered one of the key bioactive constituents, owing to its unique multi-component and multi-target advantages, it exhibits noteworthy effects including anti-tumorigenic, anti-proliferative, anti-metastatic, anti-angiogenic, anti-inflammatory, and antioxidant activities [11]. Due to its minimal toxicity and ability to target multiple regulatory pathways, CUR has demonstrated an indispensable role in the treatment and prevention of malignant tumors, it is now hailed as a third-generation anti-cancer drug within the medical community [12–15]. In China, the utilization of CUR is primarily observed within the realm of traditional Chinese medicine, according to the principles of traditional Chinese medicine, turmeric exhibits properties such as ‘alleviation of dysmenorrhea and pain’ and ‘promotion of qi and blood circulation,’ making it a commonly employed remedy for conditions like rheumatic pain and dysmenorrhea [16, 17]. In the ancient Indian Ayurveda tradition, CUR has been recognized for its potential in treating a range of human ailments, including respiratory diseases, rhinorrhea, diabetes, and rheumatism [18]. The vast repertoire of approximately 35,000 herbs exhibiting anticancer properties has led to the global recognition of phytotherapy, as indicated by data from the National Cancer Institute [19].

## Revealing the mechanism of CUR’s action against OSF

**Fig. 1:**
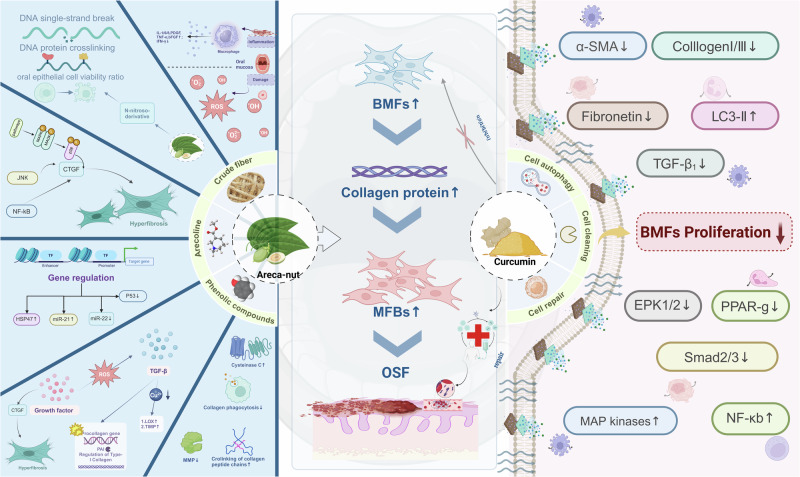
Mechanisms and pathways of action associated with oral submucous fibrosis. Mechanism analysis of curcumin in the prevention and treatment of oral submucous fibrosis, as well as the pathogenic pathways induced by arecoline. Created with BioRender. Peng (2024). BioRender.com/u13m130.

The presence of arecoline in betel nuts is the main factor causing OSF [20–22], OSF is the most common oral mucosal disease caused by habitual betel nut chewing [21], Fig. 1. In the study of the molecular mechanism of OSF, a large number of cytokines are closely associated with inflammation, oxidative stress, and fibrosis, for example, monocyte chemotaxis protein (MCP) and tumor necrosis factor α (TNFSF1A), among others, all have a significant promoting effect on the proliferation of oral fibroblasts [23]. The levels of matrix metalloproteinase (MMP-2) were found to be lower, while the expression of tissue inhibitors of metalloproteinases (TIMP-2) was increased in patients with OSF, these aberrant enzyme levels failed to effectively degrade excessive collagen in the oral mucosa, thereby impeding wound healing response and reducing collagen clearance in OSF [24].

### CUR modulates cellular autophagy to attenuate fibrosis

The findings from several studies have provided preliminary evidence indicating that CUR has the potential to modulate the progression of fibrotic diseases by regulating cellular autophagy. In the rat model of SD fibrosis that was successfully constructed and validated, Kong et al. [25] observed a significant inhibition of the epithelial-mesenchymal transition (EMT) process by CUR through enhancement of hepatocyte autophagy activity, this finding not only enhances hepatic cellular autophagy and regenerative capacity, but also elucidates the mechanisms underlying attenuation or reversal of fibrotic lesions, thereby ameliorating the fibrotic state in experimental animal models. During comprehensive investigation of OSF, a remarkable upregulation of autophagy-associated protein LC3 was observed in the affected tissues, this discovery offers a novel perspective for elucidating the pathological mechanism underlying OSF, further validation was conducted through in vitro experiments to demonstrate the potent apoptotic effects of CUR, a naturally occurring compound, on buccal mucosal fibroblasts (BMFs) by inhibiting autophagy and significantly suppressing their proliferative activity [23]. Moreover, it is noteworthy that arecoline-induced BMFs undergo a transition to more invasive myofibroblasts (MFBs), accompanied by a significant upregulation of the autophagy marker LC3-II, these findings further support the notion that autophagy may facilitate fibroblast-to-myofibroblast transition and indirectly contribute to OSF pathogenesis [26].

### CUR inhibits the activation of BMFs

The activation of BMFs is impeded by CUR, leading to a concentration-dependent decline in proliferative activity on MFBs. At lower concentrations, CUR effectively inhibits the proliferation of MFBs ; however, higher concentrations may induce cytotoxic effects and further impede cell proliferation. Furthermore, the scratch test observation revealed a notable attenuation in the migratory capacity of MFBs with increasing concentrations of CUR. This finding provides additional evidence to support the inhibitory effect of CUR on cell migration. Further investigation revealed that CUR not only impedes the proliferation and migration of activated BMFs, but also suppresses intracellular expression of various fibrosis-related proteins, including recombinant human fibronectin, α-smooth muscle actin, type I and type III collagen, among others, Fig. 1. The results of this study clearly demonstrate that CUR exhibits a significant inhibitory effect on OSF in vitro, thereby offering promising prospects for the prevention and treatment of OSF [27].

## Diverse biological activities of CUR against OSF

### Antibacterial efficacy

CUR has received approval from the U.S. Food and Drug Administration as a safe food additive with multiple pharmacological properties [28]. According to existing research, CUR has exhibited inhibitory effects against a variety of bacteria, including Staphylococcus aureus, Lactobacillus, Streptococcus, as well as certain pathogenic fungi [29–31]. Experimental evidence has demonstrated that CUR possesses the ability to bind to the cell wall and disrupt the structural integrity of bacterial cells, thereby exerting its antibacterial effects. As a standalone antibacterial agent, CUR exhibits remarkable efficacy against both Gram-negative and Gram-positive bacteria [32].

### Anti-inflammatory and anti-tumor

CUR possesses the capability to effectively suppress both acute and chronic inflammation. The anti-tumor effects of CUR involve regulating proteins that promote or inhibit cell death, growth factors like HER-2 and EGFR, matrix metalloproteinases, as well as blocking STAT3 and NF-kB signaling pathways and reducing vascular endothelial growth factor production. This ultimately reduces the release of inflammatory mediators and even allows targeted inhibition of multiple inflammatory mediators to alleviate inflammation [12]. The extracorporeal research findings demonstrate that CUR can effectively facilitate wound healing by orchestrating apoptosis of inflammatory cells during the initial stage, thereby expediting the wound healing process and abbreviating the duration of inflammation resolution [33].

### Antioxidant

CUR is a high-quality natural antioxidant, known for its ability to directly eliminate excessive oxygen free radicals in the human body, unlike synthetic antioxidants that may carry potential toxic and side effects, CUR offers a safer alternative by effectively slowing down, containing, or even removing substances caused by oxidative damage [12]. CUR possesses multiple specialized functional groups, such as the 1, 2-dihydroxy group, these structural characteristics are closely associated with diverse biological activities and demonstrate exceptional stability in antioxidant processes, thereby conferring CUR with potent antioxidant activity [34]. Currently, CUR has been utilized in the context of wound healing, Alzheimer’s disease, Parkinson’s disease, cardiovascular disease, among others [35, 36].

## The clinical application of CUR therapy in OSF

### Full-body application of CUR

According to existing literature, animal models and preliminary human trials have substantiated the safety of using CUR at a significantly higher dosage than that used in humans (12 g/d) [37]. Meanwhile, studies have confirmed that continuous intake of 6 g of CUR per day for 4–7 weeks did not observe any toxic side effects, and there were no adverse reactions even after 30 days, this indicates that CUR has safety at high doses and possesses a high level of biological safety characteristics [38]. In the clinical setting, CUR can be administered via various routes of administration, including oral ingestion, intravenous injection, and subcutaneous injection [11].

Currently, oral submucosal local injection method commonly used for the treatment of OSF can cause pain, swelling and discomfort, which may make patients reluctant to receive treatment and thus delay the condition. Oral administration method is currently the most widely accepted method of delivering drugs systemically, known for its high patient compliance and minimal postoperative pain response. However, the limited water solubility and instability of CUR under physiological conditions hinder its absorption in the human intestines upon oral administration, leading to diminished blood drug concentrations. Moreover, rapid metabolism within the body impedes CUR from fully exerting its pharmacological activity, thereby reducing its bioavailability. Consequently, these factors significantly constrain the potential clinical application of CUR [39]. Rai et al. [40] developed Turmix tablets, which consist of a combination of curcumin and piperine, as well as Turmix mouthwash. The findings demonstrated that Turmix tablets effectively ameliorated symptoms associated with OSF. Moreover, the use of Turmix mouthwash circumvented the initial hepatic metabolism of curcumin and exhibited enhanced efficacy in targeted areas, particularly in alleviating the burning sensation experienced by OSF patients. The study revealed that the incorporation of nanoparticles led to a significant augmentation in the concentration of CUR, thereby enhancing its oral bioavailability [41]. Therefore, from the perspective of enhancing the therapeutic efficacy of CUR, there is a growing research focus both domestically and internationally on developing novel CUR formulations to improve drug bioavailability, enhance drug solubility and dissolution rate, as well as optimize in vivo absorption.

### Application of CUR in the local oral cavity

In the treatment of OSF, local administration forms of CUR include capsules, tablets or lozenges, mouthwash, gels or oil formulations, etc., to achieve its effects through the oral mucosal drug delivery system. The oral mucosal drug delivery system refers to the direct absorption of drugs into the systemic circulation through the mucosa in the mouth, resulting in higher bioavailability compared to oral administration, it is commonly used for emergency treatment [42]. In general, the main administration sites for drugs in the oral mucosa are sublingual and buccal regions, among them, the sublingual mucosa is a non-keratinized mucosa within the oral cavity, characterized by its thinness and excellent drug permeability, therefore, clinically it is considered as the preferred area for rapid drug delivery. The buccal mucosa, with its relatively large surface area within the oral cavity and salivary gland duct openings, possesses excellent adhesive properties and facilitates drug administration and removal. Consequently, it serves as an ideal site for controlled-release drug delivery [43, 44].

**Fig. 2:**
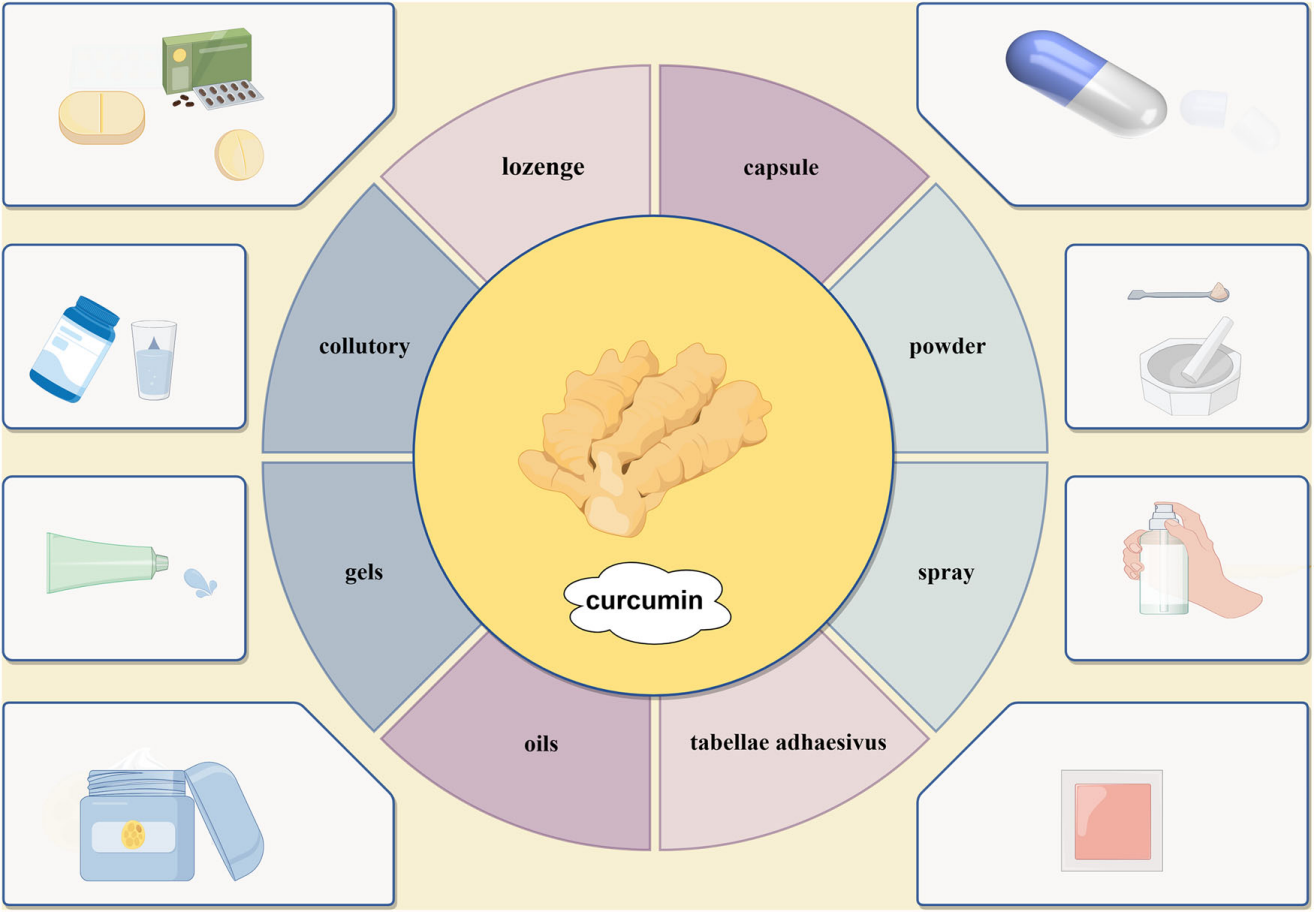
The development of curcumin formulations for the treatment of oral submucous fibrosis. In the near future, curcumin may be formulated into eight types of pharmaceutical formulations as shown in the figure. Created by figdraw.com.

The utilization of local administration via the oral mucosa for the management of OSF offers the advantages of enhanced convenience, precise targeting, and reduced drug toxicity and side effects, effectively circumventing first-pass drug metabolism [42]. In recent years, especially in the fields of CUR gel and patch formulations,etc., it has become a hot topic of research [45], Fig. 2. Chandrashekar et al. [46] developed sustained-release formulations of CUR gel and CUR mucoadhesive patches; however, oral mucosal patches offer the advantage of prolonging drug duration and improving mouth opening. According to the findings, both formulations effectively alleviated symptoms such as burning sensation and restricted mouth opening in OSF. Esposito et al. [47] developed sprayable adhesive nanoparticles containing CUR payload, thereby enhancing its aqueous solubility and enabling oral administration. Based on the aforementioned studies, it can be inferred that diverse CUR formulations hold potential for treating OSF; however, the variability in CUR administration may impact its bioavailability and consequently influence its therapeutic efficacy against OSF [48]. In recent years, researchers have actively explored numerous novel alternative therapies for the management of oral OSF, and substantial advancements have also been achieved in traditional Chinese medicine interventions such as CUR.

## Summary and outlook

In the process of OSF treatment, submucosal local injection is the predominant route for drug administration; however, most patients prefer less invasive approaches. Nevertheless, traditional injection treatments exhibit poor compliance, limited medical experience, and postoperative pain. To address these challenges and enhance patient adherence to multiple local administrations, local minimally invasive therapies like CUR have been developed to optimize recovery time after oral mucosal treatment. Nonetheless, there are several obstacles associated with using CUR including significant first-pass effect, low oral bioavailability, restricted effective absorption area in the oral mucosa, rapid drug metabolism and heterogeneous physicochemical properties. Therefore, future endeavors should focus on exploring and developing innovative pharmaceutical preparations such as microneedles or in situ gel/patch formulations loaded with nami lipid carriers spray gel or lozenges/mouthwash to overcome limitations related to curative effects stability and patient compliance compared to traditional preparations.

According to current research findings, the exploration and development of novel curcumin drugs not only holds immense potential for enhancing drug bioavailability but also enables precise targeted drug release, thereby facilitating more accurate action on lesions. This innovative approach can effectively reduce unwanted side effects and significantly improve treatment efficacy. However, there is a scarcity of clinical studies focused on augmenting the bioavailability of CUR preparations. The treatment of OSF involves complex multifactorial and multilevel pathophysiological mechanisms that necessitate interdisciplinary collaboration across fields such as biomedicine, pharmaceutical engineering, tissue engineering, genomics, and bioinformatics. Such collaborative efforts play a crucial role in deeply exploring the mechanism of action in OSF and developing new CUR preparations to prevent their progression into malignant oral diseases. It has the potential to yield groundbreaking discoveries in comprehensively studying oral disease mechanisms while making substantial contributions to human well-being.

In conclusion, despite the extensive range of treatment studies conducted both domestically and internationally on OSF, no satisfactory, efficient, and comprehensive therapeutic options have been identified thus far. Ceasing the consumption of betel nut and implementing regular patient monitoring, along with providing individualized diagnosis and treatment based on their specific circumstances, are crucial measures for the prevention and management of this disease. Although all reported drugs currently available demonstrate some efficacy in relieving symptoms of OSF, the identification of the most optimal therapeutic agents for both symptom relief and disease progression control remains elusive. The medication treatment plan and personalized treatment strategy are determined by the development trend of patients’ disease. At present, the clinical treatment methods need to be confirmed and confirmed by big data. The resolution of these scientific quandaries will hold immense significance in ensuring the elongation of patients’ lifespan and the enhancement of their quality of life.
